# The burden of nausea and vomiting during pregnancy: severe impacts on quality of life, daily life functioning and willingness to become pregnant again – results from a cross-sectional study

**DOI:** 10.1186/s12884-017-1249-0

**Published:** 2017-02-28

**Authors:** Kristine Heitmann, Hedvig Nordeng, Gro C. Havnen, Anja Solheimsnes, Lone Holst

**Affiliations:** 1 PharmacoEpidemiology and Drug Safety Research Group, School of Pharmacy, University of Oslo, Oslo, Norway; 2 Regional Medicines Information and Pharmacovigilance Centre (RELIS), Oslo, Norway; 3 Department of Global Public Health and Primary Care, University of Bergen, P-Box 7804, N-5020 Bergen, Norway

**Keywords:** Pregnancy, Morning sickness, Hyperemesis gravidarum, NVP, Burden of illness, Quality of life, Termination of pregnancy

## Abstract

**Background:**

Though nausea and vomiting is very common during pregnancy, no studies have investigated the impact of this condition on the women’s daily lives in a Scandinavian population. The aim of this study was to describe the burden of nausea and vomiting during pregnancy (NVP) on global quality of life, daily life functioning and willingness to become pregnant again according to the severity of NVP symptoms.

**Methods:**

This study is a cross-sectional population-based study conducted in Norway. Pregnant women and mothers with children <1 year of age with current or prior NVP were eligible to participate. Data were collected through an anonymous on-line questionnaire accessible from November 10^th^, 2014 to January 31^st^, 2015. Severity of NVP was measured using the 24-h Pregnancy Unique Quantification of Emesis Scale (PUQE). Associations between severity of NVP, daily life functioning and willingness to become pregnant again were tested using chi-square tests. Associations with global quality of life measured in terms of the Quality of Life Scale (QOLS) were estimated using generalized linear models and reported as unstandardized regression coefficients (β) with 95% confidence intervals (CI).

**Results:**

712 women with NVP were included in the study. NVP was significantly associated with several characteristics, including daily life functioning, quality of life and willingness to become pregnant again. The negative impact was greater the more severe the symptoms were, although considerable adverse effects were also seen among women with mild and moderate NVP symptoms. Over one fourth of the women with severe NVP considered terminating the pregnancy due to NVP, and three in four considered not to get pregnant again. Severity of NVP remained significantly associated with reduced global quality of life when adjusting for maternal characteristics and illnesses with *β* (95% CI) = −10.9 (−16.9, −4.9) for severe versus mild NVP.

**Conclusions:**

NVP as measured by PUQE had a major impact on various aspects of the women’s lives, including global quality of life and willingness to become pregnant again.

**Electronic supplementary material:**

The online version of this article (doi:10.1186/s12884-017-1249-0) contains supplementary material, which is available to authorized users.

## Background

Most pregnant women experience pregnancy-related conditions, of which nausea and vomiting during pregnancy (NVP) is by far one of the most common. Nausea affects approximately 70-80% of the pregnant population, and additional vomiting is experienced by about 50% [1–3]. The symptoms of NVP range from mild to severe, with hyperemesis gravidarum (HG) at the most severe end of the scale. HG is characterised by excessive nausea and vomiting, leading to dehydration, electrolyte and nutritional disturbances, which often necessitates hospitalisation [4, 5].

Given the high prevalence of NVP and its most often self-limiting nature, health care providers may tend to trivialise its impact [6]. Though NVP in general is not associated with increased risk of adverse pregnancy outcomes, NVP imposes significant negative impact on the women’s lives [7]. An extensive review of the literature from 1999 to 2011 included 38 studies that investigated the impact of NVP on health related quality of life and occupational, social and daily life functioning [7]. The review concluded that NVP causes decreased quality of life, and has adverse effects on social, occupational, and domestic life functioning [7]. Findings indicate that the effects of NVP are amplified with increased severity of NVP symptoms. Furthermore, an increased risk of comorbidity, especially with feelings of depression and heartburn and reflux problems has been described in the literature [8] posing an even larger burden on the women.

None of the studies included in the literature review were conducted in Norway or any other Scandinavian country. Cultural differences and differences between countries with respect to health care systems could infer that results obtained in other countries may not apply for the Norwegian pregnant population. In Norway there are approximately 60.000 births annually. The pregnancy care programme is free of charge. Furthermore Norwegian employees are entitled to sickness benefit if occupationally disabled due to own illness. The sick leave might be full time or graded (part time).

Previous studies have evaluated health related quality of life among women with NVP with generic health status measures such as the Medical Outcomes Study Short Form (SF36), SF12 (abbreviated version of SF36), and the NVP specific NVPQOL developed by Magee et al. [9–11]. Studies demonstrate great impact of NVP on health related quality of life, with increased adverse effects according to increased severity of NVP [9, 10, 12, 13]. Effects have been found on physical, social and emotional functioning, bodily pain, general health perceptions, vitality and mental health. However, health related quality of life instruments are focused on health status and do not capture how satisfied the women are with broader life domains. In specific, independence and material well-being are not captured in health status instruments [14, 15]. The Quality of Life Scale (QOLS) belongs to the global or overall quality of life tools, and is a questionnaire measuring an individual’s overall satisfaction with life using 16 questions covering relationships and material well-being, health and functioning, and personal, social and community commitment [15–18].

To the best of our knowledge, no studies have been performed with this perspective in women with NVP. Utilisation of the QOLS among women with NVP could be of great value in terms of obtaining a broader understanding of quality of life in this group. Such understanding is valuable in order to optimize pregnancy care for this patient group.

The primary aim of this study was to describe the impact of NVP on global quality of life as measured by QOLS, according to the severity of NVP symptoms as determined by PUQE. Secondary aims were to describe the impact of NVP on daily life functioning and willingness to become pregnant again according to the severity of NVP symptoms.

## Methods

This study is a cross-sectional, population-based study [19]. Pregnant women and new mothers (women with a child of < 1 year of age), who had experienced NVP during their current or last pregnancy, were invited to complete an anonymous on-line questionnaire (Additional file 1). The questionnaire was administered by SurveyXact and was accessible from 10^th^ November 2014 to 31^st^ January 2015. The questionnaire was accessible via banners with invitations to participate in the study. Banners were posted on national websites and social networks commonly visited by the pregnant population and/or new mothers (“altformamma.no”, “mamma.no”, “tryggmammamedisin.no”, “foreldre.no’s” Facebook page). The link was also posted on a Facebook page specifically created for this project, enabling the link to be shared on social media.

### Measures

The questionnaire included questions on maternal characteristics, peak severity of NVP and comorbidity. The impact of NVP was measured by a range of questions about domestic, social and occupational life functioning. The questionnaire also explored questions related to the willingness to get pregnant again. The questionnaire was reviewed by representatives from the Norwegian patient organisation for hyperemesis, Hyperemesis Norge.

### Classification of NVP, severity of symptoms

NVP was measured and classified into three groups of different severity by using the 24-h Pregnancy Unique Quantification of Emesis Scale (PUQE) [20]. PUQE consists of three items that are used to assess the severity of NVP; the number of hours of nausea, number of episodes of retching and number of episodes of vomiting within the last 24 h. Each item has five options which are scored from 1 to 5 points. The PUQE-score is calculated by adding the values from each item which adds up to a total score that ranges from 3 to 15 points. The obtained total PUQE-score was used to classify the severity of NVP into: mild ≤6 points; moderate 7–12 points; severe ≥13 points. PUQE has been validated and a significant association between PUQE and risk of hospitalisation due to severe NVP, increased healthcare costs because of NVP, reduced well-being/QOL, and inability to take iron supplements is described [21, 22]. A Norwegian translated version of PUQE that was recently validated was used in this current study [22] and is currently recommended used in routine antenatal care in Norway [23]. The version was adapted so that the women not being at the peak of their symptoms were asked to recall the extent of their NVP for a typical 24 h in the period with the most severe symptoms.

### Impact on domestic, social and occupational functioning and family planning

The women were asked about how their NVP symptoms affected their daily life. A list of potential areas NVP could impact was developed based on previous findings in the literature, and resulted in the first five items included in Table 5. The women were posed five questions about NVP’s negative impact on ability to perform domestic chores, social life, relationship with partner, ability to care for children and work capacity (as shown in Table 5), to which the women could respond “none”, “minor”, “major”, or “not relevant”. The women were also asked if they had been on sick leave due to NVP (yes/no/not relevant). In addition, the women were asked about if they ever considered terminating their pregnancy due to their NVP symptoms and if they considered not getting pregnant again due to NVP, both to which the women could respond in free text entry fields. The responses were categorised into yes, no or unsure.

### Measures of global quality of life

Global quality of life was measured by using the Quality of Life Scale (QOLS) instrument [18]. Originally, it was developed by Flanagan to measure quality of life among the general population [16-18]. The QOLS explores factors such as material comforts, health, relationships with family members and others, participation in organisations, public affairs and volunteering, socialising, work and personal development [18]. The scale was later modified by Burckhardt et al. for patients and a 16^th^ item was added – independence, ability to do for oneself [18, 24]. The women were asked to rate their current level of satisfaction with the item in question by ticking off on a seven point Likert scale ranging from very satisfied to very dissatisfied. The total QOLS score is calculated by adding up the items, and ranges from a minimum of 16 to a maximum of 112 where higher scores indicate better global quality of life.

The 16-item adapted version has been applied and validated in numerous studies across patient groups and cultures to gather quantitative information on quality of life [15, 18]. Average total score for healthy populations is approximately 90 [18]. For women in Norway the reference value for the global quality of life is a QOLS score of 85 (SD 12.3) [14]. The mean total QOLS score among women who reported having diseases or health problems in the Norwegian study was 81.0 (SD 12.8) [14]. For patients with fibromyalgia, the score ranged from approximately 70 to 73 [18, 25, 26].

A validated translated Norwegian version was applied in this current study [27]. In the validation study of the Norwegian version of the QOLS, internal consistency (Cronbach's alpha) was 0.86 and 0.89 at time 1 and 2, respectively, with a test-retest reliability of 0.83 [27].

### Potentially confounding factors

The women were asked about several socio-demographic factors and health conditions that previously have been associated with NVP. The socio-demographic variables included parity; maternal age; pre-pregnancy body mass index (BMI); smoking during pregnancy; use of folic acid; marital status; education; and working status. The variables are categorised as presented in Table 1.

**Table 1:** Maternal characteristics according to severity of NVP as defined by PUQE

	PUQE^a^				
	Total	Mild	Moderate	Severe	*p*-value^b^
	*N* = 712 n (%)	*N* = 62 n (%)	*N* = 439 n (%)	*N* = 210 n (%)	
Parity					
0 previous live births	382 (53.7)	38 (61.3)	241 (54.9)	103 (49.0)	0.185
≥ 1 previous live births	327 (45.9)	24 (38.7)	197 (44.9)	106 (50.5)	
Age, years					
Under 25	145 (20.4)	11 (17.7)	87 (19.8)	46 (21.9)	0.443
25–29	273 (38.3)	27 (43.5)	157 (35.8)	89 (42.4)	
30–39	281 (39.5)	23 (37.1)	187 (42.6)	71 (33.8)	
Over 40	13 (1.8)	1 (1.6)	8 (1.8)	4 (1.9)	
Body Mass Index (BMI) ^c^					
Underweight	33 (4.6)	2 (3.2)	23 (5.2)	8 (3.8)	0.615
Normal weight	421 (59.1)	44 (71.0)	253 (57.6)	124 (59.0)	
Overweight	139 (19.5)	10 (16.1)	86 (19.6)	42 (20.0)	
Obese	118 (16.6)	6 (9.7)	76 (17.3)	36 (17.1)	
Smoking					
No	684 (96.1)	61 (98.4)	422 (96.1)	200 (95.2)	0.557
Yes	27 (3.8)	1 (1.6)	16 (3.6)	10 (4.8)	
Use of folic acid					
Before and/or during pregnancy	673 (94.8)	57 (91.9)	419 (95.7)	197 (93.8)	0.270
No	37 (5.2)	5 (8.1)	19 (4.3)	13 (6.2)	
Marital status					
Married/cohabitating	661 (92.8)	55 (88.7)	408 (92.9)	197 (93.8)	0.363
Not married/cohabiting	51 (7.2)	7 (11.3)	31 (7.1)	13 (6.2)	
Education ^d^					
Primary or secondary	219 (30.8)	16 (25.8)	127 (28.9)	76 (36.2)	0.230
Bachelor degree	292 (41.0)	25 (40.3)	183 (41.7)	84 (40.0)	
Master degree	170 (23.9)	20 (32.3)	110 (25.1)	40 (19.0)	
Other	30 (4.2)	1 (1.6)	19 (4.3)	10 (4.8)	
Work situation					
Student	57 (8.0)	6 (9.7)	35 (8.0)	16 (7.6)	0.458
Employed	570 (80.1)	51 (82.3)	351 (80.0)	168 (80.0)	
Unemployed	53 (7.4)	1 (1.6)	32 (7.3)	19 (9.0)	
Other	32 (4.5)	4 (6.5)	21 (4.8)	7 (3.3)	

Abbreviations: *PUQE*, 24 h Pregnancy Unique Quantification of Emesis; *NVP*, nausea and vomiting of pregnancy; *OR*, odds ratio; *CI*, confidence interval

Numbers do not always add up due to missing numbers

^a^As classified by PUQE: mild: ≤6 points; moderate: 7–12 points; severe ≥13

^b^Chi-square test or Fisher’s exact test when expected count was less than 5. Adjusted for all other variables in the table

^c^Body mass index (BMI) is the weight in kilograms divided by the square of the height in metres

underweight: <18.5 kg/m^2^, normal weight: 18.5–24.9 kg/m^2^; overweight: 25.0–29.9 kg/m^2^, obese ≥30 kg/m^2^. Pre-pregnancy BMI is given

^d^Primary: ≤10 years of education (the Norwegian compulsory primary + secondary school)

secondary: 11-13 years (high school/upper secondary or vocational school)

Maternal health variables included short term illnesses and chronic diseases. A list of nine short term illnesses was presented to the women who could specify which of the illnesses they had experienced during pregnancy. These were heartburn and reflux problems, headache, constipation, common cold, pain in back, neck and/or pelvis, sleeping problems, urinary tract infection, other infections, and “other” (that could be specified in a free text entry field). The number of short term illnesses was summed up and categorised into ≤2, 3–4 and ≥5.

The women were also asked to report if they suffered from any chronic disease such as allergy, asthma, diabetes (type 1 or 2), epilepsy, cardiovascular diseases, muscle or skeletal problems, hyper-/hypothyroidism, depression/anxiety, migraine and “other”. For each chronic disease they were presented, the women could tick a box for affirmative response. The option “other” could be specified in a free text entry field. In addition the women were posed a question whether they had ever experienced any feelings of depression due to NVP while pregnant with the possibility to tick off for never, seldom, sometimes, most of the time or always.

### Statistical analyses

Descriptive statistics were utilised as appropriate. The Pearson chi-square or Fisher’s exact tests were used to assess the relationship between the severity of NVP (classified as mild, moderate and severe as defined by PUQE) on domestic, social and occupational functioning and family planning as well as socio-demographic and maternal health characteristics.

Unadjusted generalized linear models (GLM) with identity link function and normal distribution were used to analyse associations between severity of NVP (PUQE-categories) and quality of life (QOLS scores) both in the total population and in subgroups stratified according to the woman’s status at time of participation (currently pregnant, currently pregnant and experiencing NVP, new mother) to assess effect modification by sub-groups.

Since the women were asked about current quality of life and it is unlikely that NVP could affect quality of life also after birth, women who were no longer pregnant at the time of report were excluded in further analyses regarding the association between NVP and QOLS to reveal the effect of potential confounders. Firstly, unadjusted GLM models for each potential confounder were estimated. Secondly, the association between NVP and QOLS were estimated using GLM with two different levels of adjustment, and the following variables were adjusted for in each model:

Model 1: Adjustment for socio-demographic characteristics: Age, education, parity, occupational status, marital status, smoking during pregnancy, use of folic acid during pregnancy and body mass index.

Model 2: Adjustment for the same variables as in Model 1 plus additional adjustment for short term illnesses (≤2/3-4/≥5) and chronic disease (yes/no).

The potential confounders were chosen based on previously reported associations with NVP or QOLS in the literature and observed associations in the current study population. Health related variables were added as a separate step in Model 2 to observe how the association between NVP and QOLS changed after additional adjustment for co-morbidity.

The variables describing maternal characteristics were entered as categorical variables classified as shown in Table 1, with the exception of age which was entered as a continuous variable.

The residuals were assessed for normality with satisfying result for each analysis performed, and adjusted R square was reported for each model.

To further explore the relationship between severity of NVP and quality of life kernel density curves for the distribution of QOLS were fitted separately for each category of NVP including only women who were pregnant at time of participation.

All statistical analyses were performed using Statistical Package for the Social Sciences (SPSS) version 20.0 (IBM SPSS Statistics 20) for Windows (SPSS, Chicago, IL, USA).

## Results

Overall, 712 women with NVP completed the questionnaire and were included in the study.

The study participants were comparable to the general Norwegian birthing population with respect to geographic region of residence, maternal age, marital status and smoking status. A larger proportion of the study participants, however, had a higher education (B.Sc. or higher) than the women in the general birthing population, 65% vs. 47%, respectively [19].

In total, 447 (62.8%) of the 712 women were pregnant at time of participation and 265 (37.2%) were a new mother with a child < 1 year of age. As defined by PUQE, 62 (8.7%), 439 (61.7%) and 210 (29.5%) had mild, moderate and severe NVP, respectively (Table 1). There was no association between socio-demographic factors and NVP (Table 1) or having a chronic disease and NVP (Table 2). Severity of NVP however, was significantly associated with heartburn and reflux problems (71.9% among women with severe NVP vs. 58.1% among women with mild NVP) and headache (63.8% among women with severe NVP vs. 46.8% among women with mind NVP) (Table 2). Moreover, the more severe NVP symptoms, the more often the women had feelings of depression; 39.0% feeling depressed “most of the time” among women with severe NVP vs. 4.8% among women with mild NVP.

**Table 2:** Comorbidity according to NVP severity

	PUQE				
	Total	Mild	Moderate	Severe	
	*N* = 712 n (%)	*N* = 62 n (%)	*N* = 439 n (%)	*N* = 210 n (%)	*p*-value^a^
Heartburn and reflux problems	458 (64.3)	36 (58.1)	270 (61.5)	151 (71.9)	**0.02**
Headache	441 (61.9)	29 (46.8)	277 (63.1)	134 (63.8)	**0.04**
Constipation	431 (60.5)	45 (72.6)	267 (60.8)	118 (56.2)	0.07
Common cold	274 (38.4)	25 (40.3)	175 (39.9)	73 (34.8)	0.43
Pain in back, neck or pelvic	469 (65.8)	41 (66.1)	297 (67.7)	130 (61.9)	0.35
Sleep problems	450 (63.2)	33 (53.2)	282 (64.2)	134 (63.8)	0.24
Urinary tract infection	114 (15.9)	7 (11.3)	67 (15.3)	39 (18.6)	0.33
Other	91 (12.8)	9 (14.5)	60 (13.7)	22 (10.5)	0.48
Any chronic illness	345 (48.5)	21 (33.9)	221 (50.3)	103 (49.0)	0.051
Feelings of depression due to NVP					
Never	61 (8.6)	18 (29.0)	37 (8.4)	6 (2.9)	**<0.001**
Seldom	121 (17.0)	22 (35.5)	82 (18.7)	17 (8.1)	
Sometimes	323 (45.4)	19 (30.6)	211 (48.1)	93 (44.3)	
Most of the time	187 (26.3)	3 (4.8)	101 (23.0)	82 (39.0)	
Always	20 (2.8)	0 (0.0)	8 (1.8)	12 (5.7)	

^a^Chi-square test or Fisher’s exact test when expected count was less than 5. Bold *p*-values indicate statistically significant differences between the groups

**Fig. 1:**
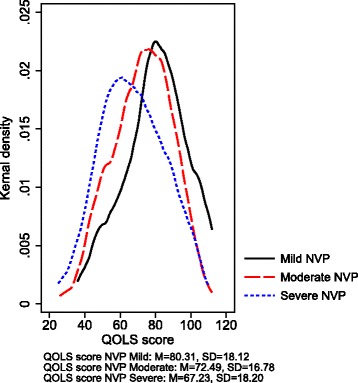
QOLS score according to NVP severity as defined by PUQE. The figure is based on analyses including only women pregnant at time of participation. QOLS score among women in the general Norwegian population was 85 [14]

In the total study population, a mean QOLS-score of 76.3 (95% CI 74.5-78.0) was found. Mean score was found to be 80.5 (95% CI 78.5-82.5) among the new mothers, 72.0 (95% CI 70.4-73.7) among women who were pregnant at time of participation and 68.1 (95% CI 66.0-70.2) among women who experienced NVP at time of participation (Table 3). Figure 1 shows the kernel density curves for the distribution of QOLS-scores according to the three groups of NVP severity among women that were currently pregnant. The distributions among women with severe and moderate NVP were shifted downwards compared to women with mild NVP.

**Table 3:** Global quality of life (QOLS) according to severity of NVP among the total population and selected sub-populations of study participants

QOLS	PUQE				
	Total	Mild	Moderate	Severe	*p*-value^a^
	Mean of total score (95% CI)	Mean of total score (95% CI)	Mean of total score (95% CI)	Mean of total score (95% CI)	
Total study population (*n* = 712)					
QOLS score	76.3 (74.5–78.0)	80.1 (75.7–84.4)	75.3 (73.7–77.0)	73.4 (71.0–75.8)	**0.03**
Pregnant at time of participation (*n* = 447)					
QOLS score	72.0 (70.4–73.7)	80.3 (74.9–85.8)	72.5 (70.6–74.4)	67.2 (63.7–70.8)	**< 0.001**
Pregnant and experiencing NVP at time of participation (*n* = 286)					
QOLS score	68.1 (66.0–70.2)	73.3 (68.9–77.6)	67.0 (64.4–69.5)	63.6 (56.2–71.0)	**< 0.001**
New mother at time of participation (*n* = 264)					
QOLS score	80.5 (78.5–82.5)	79.4 (72.2–86.6)	81.3 (78.8–83.8)	79.6 (75.9–83.2)	0.69

Abbreviations: QOLS, Quality of life scale; CI, confidence interval; PUQE, 24 h Pregnancy-Unique Quantification of Emesis. QOLS score among women in the general Norwegian population was 85 [14]

^a^ANOVA. Bold *p*-values indicate statistically significant differences between the groups

Severity of NVP symptoms was significantly associated with global quality of life among pregnant women and women experiencing NVP at time of participation, but not among the new mothers (Table 3). Lowest QOLS-score was reported by women who were pregnant and experienced severe NVP at the time of completing the questionnaire (63.6, 95% CI 56.2-71.0) (Table 3).

In adjusted analyses including only pregnant women (Table 4), increased NVP severity was significantly associated with reduced QOLS score after adjustment for maternal characteristics in Model 1 with β (95% CI) = −11.7 (−17.6, −5.7) for severe NVP compared with mild NVP. The association was slightly reduced after adjustment for short term illnesses and chronic disease in Model 2, but was still significant with β (95% CI) = −10.9 (−16.9, −4.9).

**Table 4:** Regression analyses for the association between severity of NVP and global quality of life controlling for demographic variables, and comorbidity. (*N* = 447)

	Univariate models		Model 1		Model 2	
	Unstandardized regression coefficients (β) (95% CI)	*p*-value	Unstandardized regression coefficients (β) (95% CI)	*p*-value	Unstandardized regression coefficients (β) (95% CI)	*p*-value
Severity of NVP						
Mild	Ref.		Ref.		Ref.	
Moderate	–7.8 (–13.3,–2.4)	**0.005**	–6.7 (–12.0,–1.4)	**0.014**	–5.9 (–11.2,–0.5)	**0.032**
Severe	–13.1 (–19.1,–7.0)	**<0.001**	–11.7 (–17.6,–5.7)	**<0.001**	–10.9 (–16.9,–4.9)	**<0.001**
Parity						
0 previous live births	Ref.		Ref.		Ref.	
≥ 1 previous live births	–4.7 (–7.9,–1.4)	**0.005**	–6.6 (–10.0,–3.1)	**<0.001**	–6.7 (–10.2,–3.2)	**<0.001**
Age, years	0.3 (–0.1, 0.6)	0.100	0.2 (–0.2, 0.6)	0.299	0.2 (–0.2, 0.6)	0.320
Body Mass Index (BMI) ^a^						
Underweight	–4.1	0.2	–2.4 (–10.5, 5.8)	0.565	–1.7 (–9.9, 6.4)	0.677
Normal weight	Ref.		Ref.		Ref.	
Overweight	–2.7	0.1	–3.6 (–7.8, 0.5)	0.087	–4.0 (–8.2, 0.1)	0.056
Obese	–3.7	**0.045**	–5.6 (–10.1,–1.1)	**0.014**	–5.5 (–9.9,–1.0)	**0.016**
Smoking						
No	Ref.		Ref.		Ref.	
Yes	2.2 (–6.4, 10.7)	0.617	6.0 (–2.5, 14.4)	0.166	5.9 (–2.5, 14.3)	0.168
Use of folic acid						
No	Ref.		Ref.		Ref.	
Yes, before and/or during pregnancy	1.8 (–5.5, 9.0)	0.634	–1.4 (–8.4, 5.6)	0.696	–1.1 (–8.1, 5.9)	0.762
Marital status						
Married/cohabitating	Ref.		Ref.		Ref.	
Not married/cohabiting	–6.0 (–11.9,–0.1)	**0.046**	–6.8 (–12.6,–1.0)	**0.021**	–6.9 (–12.7,–1.1)	**0.019**
Education ^b^						
Primary or secondary	–8.5 (–12.9,–4.1)	**<0.001**	–6.1 (–11.0,–1.2)	**0.014**	–6.1 (–11.0,–1.2)	**0.015**
Bachelor degree	–6.4 (–10.5,–2.3)	**0.002**	–5.1 (–9.2,–1.0)	**0.015**	–5.0 (–9.1,–0.8)	**0.018**
Master degree	Ref.		Ref.		Ref.	
Other	–14.2 (–22.3,–6.1)	**0.001**	–13.0 (–21.2,–4.8)	**0.002**	–12.8 (–21.0,–4.6)	**0.002**
Work situation						
Student	–4.1 (–10.3, 2.1)	0.198	–2.6 (–8.8, 3.6)	0.407	–3.3 (–9.5, 2.9)	0.290
Employed	Ref.		Ref.		Ref.	
Unemployed	–3.3 (–10.2, 3.6)	0.344	3.3 (–3.9, 10.5)	0.366	4.2 (–3.0, 11.4)	0.256
Other	0.9 (–6.7, 8.5)	0.815	2.9 (–4.4, 10.3)	0.436	4.4 (–3.0, 11.8)	0.245
Number of short term illnesses						
≤ 2	Ref.		–	–	Ref.	
3–4	–4.2 (–8.4, 0.1)	0.056			–3.2 (–7.3, 1.0)	0.133
≥ 5	–6.2 (–10.7,–1.8)	**0.007**			–5.5 (–9.9,–1.1)	**0.014**
Chronic disease						
No	Ref.		–	–	Ref.	
Yes	–2.2 (–5.5, 1.1)	0.184			–2.2 (–5.4, 1.1)	0.187
*Adjusted R square*			0.10		0.11	

Abbreviations: *Ref* = reference category. Models were estimated using generalized linear models with QOLS as dependent variable, identity link function and assuming a normal distribution of QOLS. Only women who were currently pregnant are included in the analyses. Bold *p*-values indicate significant differences between the groups

^a^Body mass index (BMI) is the weight in kilograms divided by the square of the height in metres; underweight: <18.5 kg/m^2^, normal weight: 18.5–24.9 kg/m^2^; overweight: 25.0–29.9 kg/m^2^, obese ≥30 kg/m^2^

underweight:

^b^Primary: ≤10 years of education (the Norwegian compulsory primary + secondary school), secondary: 10–12 years (high school/upper secondary or vocational school)

NVP greatly interfered with the women’s daily lives and was found to have important adverse effects on daily life functioning (Table 5). More than 70% of the women in the total study population experienced that NVP had a major adverse impact on taking care of household chores and on social life functioning. As many as 63.5% reported that NVP had a major adverse effect on the ability of care for their children, and for approximately 80% the relationship with partner was negatively affected to some extent. Work capacity was impaired due to NVP for most of the women, and 428 (60.1%) had been on sick leave due to NVP. Family planning was also affected, especially among women with severe symptoms of which 75.7% of the women considered not to get pregnant again. A total of 26.7% of the women with severe symptoms considered to terminate the pregnancy due to NVP.

**Table 5:** Negative impact of NVP on daily life functioning and willingness to get pregnant again

	PUQE				
	Total^b^	Mild	Moderate	Severe	*p*-value^a^
	*N* = 712 n (%)	*N* = 62 n (%)	*N* = 439 n (%)	*N* = 210 n (%)	
1. Taking care of household chores					
No impact	29 (4.1)	14 (22.6)	14 (3.2)	1 (0.5)	**<0.001**
Minor impact	150 (21.1)	37 (59.7)	103 (23.5)	10 (4.8)	
Major impact	533 (74.9)	11 (17.7)	322 (73.3)	199 (94.8)	
2. Social life					
No impact	44 (6.2)	18 (29.0)	23 (5.2)	3 (1.4)	**<0.001**
Minor impact	155 (21.8)	34 (54.8)	103 (23.5)	17 (8.1)	
Major impact	513 (72.1)	10 (16.1)	313 (71.3)	190 (90.5)	
**3**. Relationship with partner ^c, n=682^					
No impact	134 (19.6)	23 (38.3)	77 (18.2)	34 (17.1)	**<0.001**
Minor impact	306 (44.9)	32 (53.3)	193 (45.7)	80 (40.2)	
Major impact	242 (35.5)	5 (8.3)	152 (36.0)	85 (42.7)	
4. Ability to care for children ^d, n=351^					
No impact	31 (8.8)	10 (32.3)	16 (7.7)	5 (4.5)	**<0.001**
Minor impact	97 (27.6)	17 (54.8)	67 (32.2)	13 (11.6)	
Major impact	223 (63.5)	4 (12.9)	125 (60.1)	94 (83.9)	
5. Work capacity ^e, n=671^					
No impact	28 (4.2)	8 (13.8)	20 (4.9)	0 (0.0)	**<0.001**
Minor impact	147 (21.9)	40 (69.0)	95 (23.2)	12 (5.9)	
Major impact	496 (73.9)	10 (17.2)	294 (71.9)	191 (94.1)	
6. Sick leave due to NVP ^f, n=649^					
No	221 (34.1)	50 (84.7)	153 (38.8)	18 (9.2)	**<0.001**
Yes	428 (65.9)	9 (15.3)	241 (61.2)	178 (90.8)	
7. Considered to terminate the pregnancy due to NVP					
No	608 (85.5)	62 (100.0)	393 (89.5)	153 (72.9)	**<0.001**
Yes	100 (14.1)	0 (0.0)	44 (10.0)	56 (26.7)	
Unsure	3 (0.4)	0 (0.0)	2 (0.5)	1 (0.5)	
8. Consider not to get pregnant again					
No	333 (46.8)	54 (87.1)	233 (53.1)	46 (21.9)	**<0.001**
Yes	353 (49.6)	6 (9.7)	188 (42.8)	159 (75.7)	
Unsure	25 (3.5)	2 (3.2)	18 (4.1)	5 (2.4)	

Abbreviations: *CI*, confidence interval; *PUQE*, 24 h Pregnancy-Unique Quantification of Emesis; *NVP*, nausea and vomiting of pregnancy

^a^Chi-square test or Fisher’s exact test when expected count were less than 5. Bold *p*-values indicate statistically significant differences between the groups

^b^The sums do not always add up to total due to missing (“not relevant”)

^c^3.8% responded “not relevant”

^d^52.3% did not have any children

^e^6.8% responded “not relevant”

^f^8.8% responded “not relevant”

Severity of NVP was significantly associated with impaired ability to engage in domestic, occupational and social activities, with increased impact according to increased severity (Table 5). More women with severe symptoms reported major impact on the various parameters, however it is worth noticing that a large proportion of women with moderate symptoms reported major adverse impact on domestic, social and occupational functioning.

## Discussion

Several of the findings are important for clinical practice. Global quality of life was significantly reduced according to severity of NVP. The study also shows that NVP has impact on daily life functioning and willingness to become pregnant again. This study is the first study to assess the impact of NVP on these outcomes in a Scandinavian population.

Previous studies have found major impacts of NVP on health related quality of life [7]. This current study also demonstrates that NVP has adverse effects on the global quality of life measured by the QOLS. The total QOLS score among women who were pregnant or who experienced NVP at time of participation were 72 and 68, respectively. This is low compared to other populations such as women in the general Norwegian population with an average score of 85 [14]; patients with various chronic diseases such as rheumatic disease groups, psoriasis and chronic obstructive pulmonary disease, who score above 80 on the QOLS [18]; fibromyalgia patients with scores around 70–73 [18, 25, 26]; and Israeli patients with posttraumatic stress disorder with a score of 61 [18]. Furthermore, we found that global quality of life was significantly associated with the severity of NVP. The mean total QOLS score among women with severe NVP symptoms that were pregnant or were experiencing NVP at time of participation was 67 and 64, respectively, demonstrating that severe NVP affects global quality of life to a great extent. However, when only the new mothers were included in the analyses, no association with severity of NVP was detected. Furthermore, the mean QOLS score among the new mothers was above 80, and higher than for the two other sub-groups, approaching that of the general Norwegian population [14]. This is reassuring as it may imply that the severity of NVP experienced while pregnant does not affect quality of life after birth, and that global quality of life normalises after birth for most women, despite having suffered from severe NVP while pregnant.

We also investigated whether the inclusion of potential confounders had an impact on the association between NVP and QOLS for pregnant women. The adjusted analysis demonstrated that the association could not be explained by the investigated factors.

Major impact of NVP on the women’s quality of life has also previously been demonstrated in studies from other countries. In line with our results, the effects have been found to be increased according to the severity of the NVP symptoms [10, 12, 13]. Women suffering from more severe symptoms were found to have a physical quality of life close to that among women with breast cancer, and a mental quality of life comparable to that seen among women with postpartum depression [10].

In addition, this study demonstrates that NVP affects the women’s daily life functioning and willingness to become pregnant again. Especially for the women with severe symptoms the adverse effects of NVP were found to be substantial. The fact that more than one fourth of the women with severe symptoms reported that they had considered terminating the pregnancy, is highly concerning. Other studies conducted among women with HG report that 15% of the women actually had terminated a pregnancy due to the severity of their symptoms [28]. A Canadian study also demonstrated that termination of pregnancy occurs due to NVP [29].

It is also worth noting that among women with severe symptoms, 76% reported that they considered never to get pregnant again, 84% reported that the NVP had major adverse effects on the ability to care for their children, and 43% reported major impact on the relationship with their partner, reflecting substantial effects on family life functioning. In total 94% reported major impact on their work capacity and over 90% had been on sick leave due to NVP, illustrating that occupational functioning is affected for most women with severe NVP. However, considerable adverse effects were also seen among women with moderate symptoms, and even some women with mild symptoms reported major impact on different aspects of daily life functioning. This is in line with other studies describing that even mild NVP affected important part of the women’s daily lives, such as caring for children, relationship with partner, work productivity and intent to become pregnant again [13, 30].

Our study implies that adequate management of NVP is not only important for moderate to severe cases, but also for mild cases. Health care providers should perform individual assessments of the degree of NVP and its implications on the woman’s quality of life and daily life functioning for each woman presenting with NVP.

### Strengths and limitations

This study has several strengths and limitations that need to be addressed. A conventional response rate could not be calculated due to the web-based design of the study. Furthermore, a self-selection bias of more motivated women cannot be ruled out, possibly explaining the relatively high proportion of women with severe NVP and thus leading to an over-estimation of the amount of women with severe symptoms. However, the utilisation of the internet for recruitment purposes and collection of data enabled a high number of women to be reached from all over Norway. The participants in this study were reasonably comparable to the general birthing population in Norway, with a strikingly similar geographic spread, with the exception of the somewhat higher rate of education among the participants [19]. This may be due to the high internet penetration in Norway. In total, 97% of the women aged 16–44 in Norway use internet on a daily basis [31], which may infer that this methodology is especially appropriate for the target population of this study. There is an increased use of the internet for research purposes [32]. Web-based recruitment has shown reasonable validity in epidemiological studies [33, 34]. Furthermore, the information reported in web-based questionnaires has similar quality to that reported in paper based questionnaires [35–37].

Our results rely on the women’s accurate recall of the consequences experienced due to NVP. As most women were past the peak of NVP severity (94%), maternal reporting of peak NVP severity was retrospective. This may have introduced a risk for overestimation of the NVP symptoms as shown by Koren et al. [38]. As women with severe symptoms might be overrepresented, this may have biased our results towards more severe consequences for the group as a whole. However, as we have information about the severity of the NVP symptoms, the results are presented according to the severity of NVP.

## Conclusion

Global quality of life was significantly reduced according to severity of NVP. Severity of NVP was also significantly associated with negative effects on various aspects of daily life functioning and willingness to become pregnant again. Health care providers should be aware of the high burden NVP represents for the women, and provide the necessary support and care needed in each individual case. Prospective studies are needed to determine whether earlier treatment of women with NVP may reduce the need for sick leave, the risk of progression into severe symptoms and hospitalisation and reduce the adverse effects of NVP on the women’s lives.

## Additional file

Additional file 1: Questionnaire. (PDF 82 kb)

## Abbreviations

BMI: Body mass index
GLM: Generalised linear model
HG: Hyperemesis gravidarum
NVP: Nausea and vomiting during pregnancy
PUQE: 24 h Pregnancy-unique quantification of emesis-scale
QOLS: Quality of life scale
SPSS: Statistical Package for Social Sciences
