# Supplementary material for: The burden of nausea and vomiting during pregnancy: severe impacts on quality of life, daily life functioning and willingness to become pregnant again – results from a cross-sectional study
Source: BMC Pregnancy Childbirth. 2017 Feb 28;17:75. doi: 10.1186/s12884-017-1249-0 (PMC5329925; doi:10.1186/s12884-017-1249-0)
Supplement: Additional file 1: — Questionnaire. (PDF 82 kb) [file 12884_2017_1249_MOESM1_ESM.pdf]

The burden of nausea and vomiting during pregnancy: severe impacts on quality of life, daily life functioning and willingness to become pregnant again - results from a cross-sectional study
Kristine Heitmann, M.Sc.; Hedvig Marie Egeland Nordeng, Dr.Philos.; Gro Cecilie Havnen, M.Sc.; Anja Solheimsnes, M.Sc.; Lone Holst, PhD BMC Pregnancy and Childbirth

Questionnaire

1. About yourself

1.1 In which year were you born?
□ 1996 □ 1995 □ 1994 …. □ 1965 □1955 □1954

1.2 In which municipality do you live?

☐ Akershus
☐ Rogaland
☐ Sogn og Fjordane
☐ Sør-Trøndelag
☐ Telemark
☐ Troms
☐ Vest-Agder
☐ Vestfold
☐ Østfold
☐ Annet:____________________
☐ Oslo
☐ Aust-Agder
☐ Buskerud
☐ Finnmark
☐ Hedmark
☐ Hordaland
☐ Møre og Romsdal
☐ Nordland
☐ Nord-Trøndelag
☐ Oppland

1.3 What is your marital status?
□ Married/living together with partner □ Single □ In a relationship □ Other

1.4 What is the highest degree you obtained?
□ 9/10 years elementary school □ 3-year high school □ University college □ University □ Other education

1.5 What is your current work situation?
□ Student □ Working (Check here if you are employed but on sick or maternity leave) □ Stay-home mom
□ Other, please specify:__________________

1.6 Did you smoke during your pregnancy AFTER you found out you were pregnant?
□ Yes, I smoked regularly
□ Yes, occasionally
□ No, never

1.7 Do you have any chronic diseases?
□ Allergies □ Asthma □ Diabetes (type 1 or 2) □ Epilepsy □ Cardiovascular disease □ Musculosceletal disorders □ Hypo- or hyperthyroidism □ Depression/ anxiety □ Migraine □ Other: _________ □ None

1.8 Weight before pregnancy
If you are not able to answer this question, please write "do not know" or "?”

1.9 Height
If you are not able to answer this question, please write "do not know" or "?”

2. Pregnancy

2.1 Are you pregnant now?
□ Yes □ No

(If yes) In which week of pregnancy are you now?
Week _______

(If yes) Are you pregnant with more than one fetus?
□ No □ Yes, with twins or triplets □ Do not Know

(If yes) What is your current weight?
___________kg

(If yes) How many children do you have already?
No □ 1 □ 2 □ 3 □ 4 □ 5 □ more than 5

(If yes) We ask you to answer the rest of the questionnaire on the basis of your experiences from this pregnancy

(If no) How old is your youngest child?
__________weeks or ______________months

(If no) Were you pregnant with more than one fetus in the last pregnancy?
□ No □ Yes, with twins or triplets

(If no) How many children do you have in total?
□1 □2 □3 □4 □5 □more than 5

(If no) We ask you to answer the rest of the questionnaire on the basis of your experiences from your last pregnancy

3. Nausea and vomiting of pregnancy during your current/last pregnancy

3.1 In which pregnancy week did your nausea start? (Calculated from the first day of the last menstrual period before your pregnancy)
□ Week 1 □ Week 2 □ Week 3 ... .. □ Week 39 □ Week 40 □ later than week 40

3.2 (If yes during 2.1)
Do you suffer from nausea/vomiting right now?
□ Yes □ No
(If yes) In the last 24 hours:
I. For how long have you felt nauseated or sick to your stomach?
□ Not at all □1 hour or less □ 2-3 hours □ 4-6 hours □ More than 6 hours
II. Have you vomited or thrown up?
□ 7 or more times □ 5-6 times □ 3-4 times □ 1-2 times □ I did not throw up
III. How many times have you had retching or dry heaves without bringing anything up?
□ No time □ 1-2 times □ 3-4 times □ 5-6 times □ 7 or more times

How many hours have you slept out of 24 hours?
□ 1 □ 2 □ 3 ...□ 11 □ 12 □ More than 12
Are you at the peak of your nausea symptoms?
□ Yes □ No

(If new mom or symptoms not peaking at this time point) Please think back on a typical 24 hours during the period when your symptoms were peaking:
I. For how long did you feel nauseated or sick to your stomach?
□ Not at all □ 1 hour or less □ 2-3 hours □ 4-6 hours □ More than 6 hours
II. Did you vomit or throw up?
□ 7 or more times □ 5-6 times □ 3-4 times □ 1-2 times □ I did not throw up
III. How many times did you have retching or dry heaves without bringing anything up?
□ No time □ 1-2 times □ 3-4 times □ 5-6 times □ 7 or more times
How many hours did you sleep out the 24 hours?
□ 1 □2 □3…..□ 11 □ 12 □ More than 12

(If no) In which pregnancy week did your nausea and vomiting of pregnancy stop? (Calculated from the first day of the last menstrual period before your pregnancy)
□ Prior to week 10 □ week 10 □ week 11 ….□ week 39 □ week 40 □ later than week 40

4. Nausea and vomiting of pregnancy in everyday life

We also want to get some information on how nausea and vomiting of pregnancy influenced your everyday life

If you are pregnant now, please answer for your current pregnancy only.
If you are the mother of young children under 1 year, please answer for your last pregnancy.

4.1 What effect would you say that the nausea and vomiting of pregnancy had on your everyday life with regards to the following aspects?
Inhibition of the ability to take care of household chores:
□ None □ Minor □ Major
Reduced social life
□ None □ Minor □ Major
Negative impact on the relationship with your partner
□ None □ Minor □ Major □ Not applicable
The ability to care for any children from previous pregnancies
□ None □ Minor □ Major □ I do not have children from before
Reduced work capacity
□ None □ Minor □ Major □ Not applicable
Do you/Have you experienced any feelings of depression as a result of your nausea and vomiting of pregnancy?
□ Never □ Rarely □ Sometimes □ Often □ Always
4.2 Have you ever considered terminating your pregnancy due to your nausea and vomiting of pregnancy?

____

4.3 Have you ever considered not having more children due of your nausea and vomiting of pregnancy?

____

5. Pregnancy-associated issues

If you are pregnant now, please answer for your current pregnancy only.
If you are the mother of young children under 1 year, please answer for your last pregnancy.

5.1 Have you experienced any of the following issues during your pregnancy?

□ Acid reflux / heartburn □ Constipation □ Colds □ Headaches □ Pain in the back / neck / pelvic
□ Sleeping problems □ Urinary tract infection □ Other infections: ___________________ □ Other
□ No symptoms whatsoever

5.2 Have you taken any of the following during your pregnancy (not necessarily for nausea)?

Folic acid / folate: ☐ Yes, before I was pregnant ☐ Yes, during my pregnancy ☐ No ☐ I do not remember

Iron supplements: ☐Yes, before I was pregnant ☐Yes, during my pregnancy ☐ No ☐I do not remember

6. Nausea and vomiting of pregnancy and sick leave

If you are pregnant now, please answer for your current pregnancy only.
If you are the mother of young children under 1 year, please answer for your last pregnancy.

6.1 If you are / were working; have you been on sick leave because of your nausea and vomiting of pregnancy?

□ Yes □ No □ I do not remember □ Not applicable

(If yes) Were you on a full-time sick leave or partially?

□ Full-time sick leave □ Partially □ Both

(If yes) Estimate the number of days you were on sick leave in total (merge partial days so that e.g. two days with 50% sick leave are considered one full day)
If you are not able to answer this questions, please write "I do not know" or "?"

____

(If yes) Who initiated the sick leave?

□ Physician □ Myself □ Other, who?: _____________ □ I do not remember

7. Quality of life

7.1 We will now ask you to read through every item, and tick off for the alternative that best describes how satisfied you are at the moment.

It is important that you answer every item, even if you don't participate in the activity or having the relationship. You may be satisfied or dissatisfied with not taking part in the activity or having the relationship.							
	**Terrible**	**Unhappy**	**Mostly dis-satisfied**	**Mixed**	**Mostly satisfied**	**Pleased**	**Delighted**
**Material comforts;** home, food, modern conveniences, financial security							
**Health;** being physically fit and vigorous							
**Relationships with parents, sibling and other relatives;** communicating, visiting, helping							
**Having and rearing children**							
**Close relationship with spouse or significant other**							
**Close friends**							
**Helping and encouraging others, participating in organizations, volunteering**							
**Participating in political organizations or public affairs**							
**Learning;** Attending school, improving knowledge							
**Understanding yourself;** Knowing what life is about							
**Work;** Job or home							
**Expressing yourself creatively**							

	**Terrible**	**Unhappy**	**Mostly dis-satisfied**	**Mixed**	**Mostly satisfied**	**Pleased**	**Delighted**
**Socializing;** Meeting other people, doing things							
**Reading, music, or watching entertainment**							
**Participating in active recreation**							
**Independence;** Being able to do things yourself							
